# Supplementary material for: Sleep Duration is Inversely Associated with Serum Uric Acid Concentrations and Uric Acid to Creatinine Ratio in an Elderly Mediterranean Population at High Cardiovascular Risk
Source: Nutrients. 2019 Apr 1;11(4):761. doi: 10.3390/nu11040761 (PMC6521014; doi:10.3390/nu11040761)
Supplement: Supplementary file 1 [file nutrients-11-00761-s001.pdf]

Supplemental Table S1. Spearman's correlation analysis between uric acid, creatinine, and leukocytes.

	Uric acid	Creatinine	Leukocytes
Uric acid	1		
Creatinine	0.37 *	1	
Leukocytes	0.11 *	0.12 *	1

* p value < 0.05.
